# Development of Droplet Microfluidics Enabling High-Throughput Single-Cell Analysis

**DOI:** 10.3390/molecules21070881

**Published:** 2016-07-05

**Authors:** Na Wen, Zhan Zhao, Beiyuan Fan, Deyong Chen, Dong Men, Junbo Wang, Jian Chen

**Affiliations:** 1 Institute of Electronics, Chinese Academy of Sciences, Beijing 100190, China; wenna14@mails.ucas.ac.cn (N.W.); zhaozhan@mail.ie.ac.cn (Z.Z.); fanbeiyuan@ucas.ac.cn (B.F.); dychen@mail.ie.ac.cn (D.C.); 2 Wuhan Institute of Virology, Chinese Academy of Sciences, Wuhan 430071, China; d.men@wh.iov.cn

**Keywords:** droplet microfluidics, single-cell encapsulation, single-cell proteomic analysis, single-cell genetic analysis, single-cell screening, high-throughput

## Abstract

This article reviews recent developments in droplet microfluidics enabling high-throughput single-cell analysis. Five key aspects in this field are included in this review: (1) prototype demonstration of single-cell encapsulation in microfluidic droplets; (2) technical improvements of single-cell encapsulation in microfluidic droplets; (3) microfluidic droplets enabling single-cell proteomic analysis; (4) microfluidic droplets enabling single-cell genomic analysis; and (5) integrated microfluidic droplet systems enabling single-cell screening. We examine the advantages and limitations of each technique and discuss future research opportunities by focusing on key performances of throughput, multifunctionality, and absolute quantification.

## 1. Introduction

Cellular heterogeneity arising from stochastic expressions of genes, proteins and metabolites is a key component of cell biology and, thus, analyzing individual cells can better reveal cell-to-cell variations which are masked by bulk measurements [1,2]. Technique developments in single-cell separation (e.g., liquid chromatography and electrophoresis) and detection (e.g., laser induced fluorescence, mass spectroscopy imaging, electrochemistry, and chemiluminescence) enable single-cell analysis at both the intracellular levels (e.g., genomic, transcriptomic, and proteomic studies) and at the levels of secretory responses, microenvironments, and cell-cell interactions [3,4,5,6].

One emerging tool with the potential to provide new opportunities of single-cell analysis is microfluidics [7,8,9]. As a technology on the processing and manipulation of small amounts of fluids (10^−9^ to 10^−18^ liters) in channels with dimensions of tens of micrometers [10,11,12], microfluidics matches with the sizes of biological cells and, thus, functions as a promising platform for cellular analysis [12,13,14], enabling the characterization of biochemical (e.g., gene [15] and protein [16,17,18]) and/or biophysical properties (mechanical [19,20,21] and electrical properties [19,22,23]) of single cells.

As a subset of microfluidics, droplet microfluidics involves the production of microscale droplets (typically on the order of tens to hundreds of µm in diameter) of one fluid within a second immiscible carrier fluid in a high-throughput manner, leading to promising applications in a variety of fields including directed evolution, tissue printing, and polymerase chain reaction (PCR) [24,25,26,27,28,29]. In the field of single-cell analysis, droplet microfluidics is also promising since each cell can be individually confined within its own droplet, and cell-secreted molecules can rapidly reach detectable concentrations in the confined fluid surrounding the encapsulated single cells [30,31,32,33].

Rather than a comprehensive review, this mini review is aimed to briefly summarize key developments in the applications of droplet microfluidics in single-cell analysis, which includes (1) prototype demonstration of single-cell encapsulation in microfluidic droplets; (2) technical improvements of single-cell encapsulation in microfluidic droplets; (3) microfluidic droplets enabling single-cell proteomic analysis; (4) microfluidic droplets enabling single-cell genomic analysis; and (5) integrated microfluidic droplet systems enabling single-cell screening. Furthermore, we examine the advantages and limitations of each technique and discuss future research opportunities by focusing on three key parameters: throughput, multifunctionality, and absolute quantification.

## 2. Prototype Demonstration of Single-Cell Encapsulation in Microfluidic Droplets

**Figure 1:**
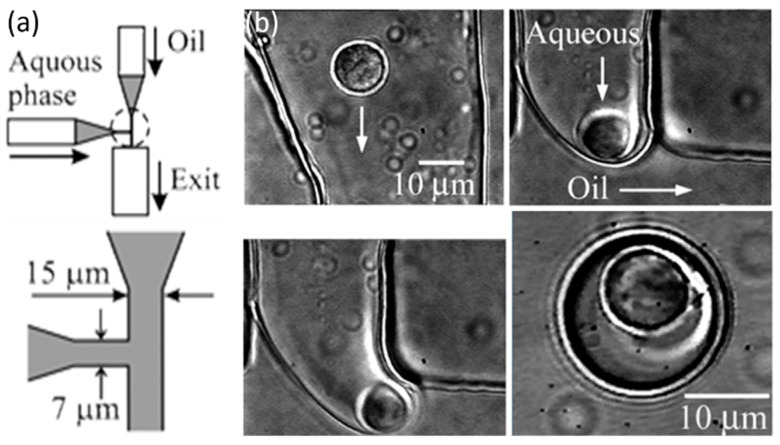
(**a**) Schematic of a microfluidic T channel and (**b**) sequences of images showing the encapsulation of a single B lymphocyte into an aqueous droplet in silicone oil. Optical trapping was used to transport and position the cell close to the water/oil interface. Upon application of a pressure pulse to the microchannels, the cell was carried away by the flow as the droplet was sheared off. Reproduction with permission from [34].

As pioneers in the field of single-cell encapsulation in microfluidic droplets, in 2005, Chiu et al. combined optical trapping and microfluidic T-junctions to encapsulate single cells in picoliter- or femtoliter-volume aqueous droplets surrounded by an immiscible phase (see Figure 1). More specifically, optical trapping was used to transport and position single cells close to the water/oil interface, followed by a pressure pulse, which sheared off the aqueous phase into a single droplet with single cells encapsulated. In addition, laser-induced lysis of cells within droplets was demonstrated and the activities of an intracellular enzyme were assayed using corresponding fluorogenic substrates [34].

In addition to confining single cells in aqueous droplets surrounded by a continuous oil phase, microfluidic flow-focusing structures were also used to (1) encapsulate single cells (e.g., Hela, MCF7, and yeast cells) in lipid vesicles surrounded by a continuous aqueous phase [35] and to (2) generate cell-enclosing agarose microcapsules in a continuous oil phase [36]. Although these prototype devices suffered from key shortcomings, such as low throughputs and low single-cell trapping efficiencies, these pioneering studies indeed open new possibilities for carrying out single-cell studies in droplet microfluidics, and pave the foundations for the upcoming studies focusing on single-cell proteomic analysis, genetic analysis, and high-throughput screening based on droplet microfluidics.

## 3. Technical Improvements in Single-Cell Encapsulation in Microfluidic Droplets

Accurate control of the number of cells per droplet is a challenging issue due to the nature of Poisson (random) encapsulation where the Poisson probability of a droplet containing one and only one cell is only 36.8%, and the probability of pairing two distinct cell types in a droplet is reduced to 13.5%. In practice, in order to make sure that no two cells are confined within one droplet, cell suspensions are further diluted, leading to a large number of empty droplets, which is wasteful [33].

**Figure 2:**
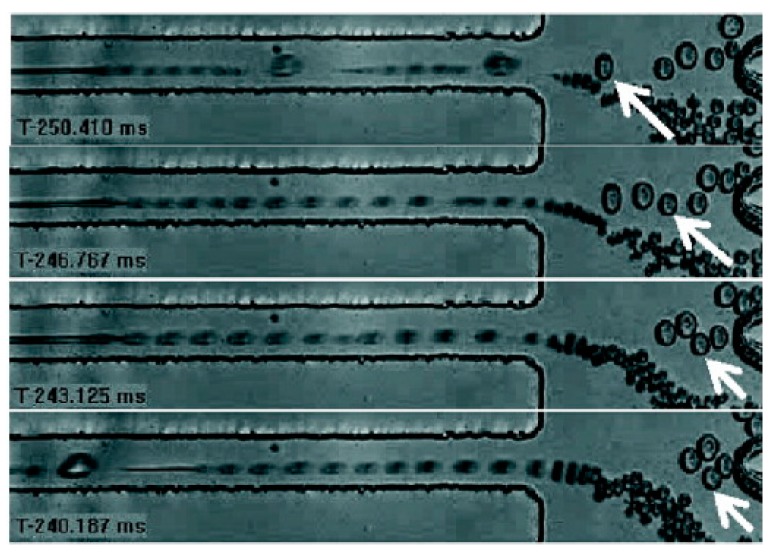
Size-based droplet sorting after cellular encapsulation. A purely hydrodynamic approach for single-cell encapsulation, followed by spontaneous self-sorting of these droplets based on lateral drift of deformable objects in a shear flow, and sterically-driven dispersion in a compressional flow. Reproduction with permission from [37].

The first approach to address this issue was to remove empty droplets after single-cell confinements [37,38,39,40]. As the first demonstration, Viovy et al. presented a purely hydrodynamic method for encapsulation of single cells into picoliter droplets, followed by spontaneous self-sorting based on the sizes. As shown in Figure 2, encapsulation was realized based on a cell-triggered Rayleigh–Plateau instability in a flow-focusing geometry, and self-sorting relied on two extra hydrodynamic mechanisms, which are lateral drift of deformable objects in a shear flow, and sterically-driven dispersion in a compressional flow, respectively. Successful encapsulation and sorting of 70%–80% of the droplets containing one and only one cell was reported, demonstrating a significant improvement in comparison to random cell encapsulation [37].

Furthermore, passive separation of microfluidic droplets by size was also enabled by deterministic lateral displacement where a tilted pillar array allows droplets smaller than a certain critical diameter to follow the direction of the incoming fluid flow while larger droplets are constrained to follow the tilted lanes of the pillar array [39,40]. Based on this microfluidic structure, recent studies (1) sorted out shrunken yeast-cell containing droplets from 31% larger diameter droplets which were generated at the same time containing only media [39]; and (2) separated large droplets encapsulating tumor cells (diameter, ~25 µm) and small empty droplets (diameter, ~14 µm), enriching the single-cell encapsulated droplets to roughly 78% [40].

**Figure 3:**
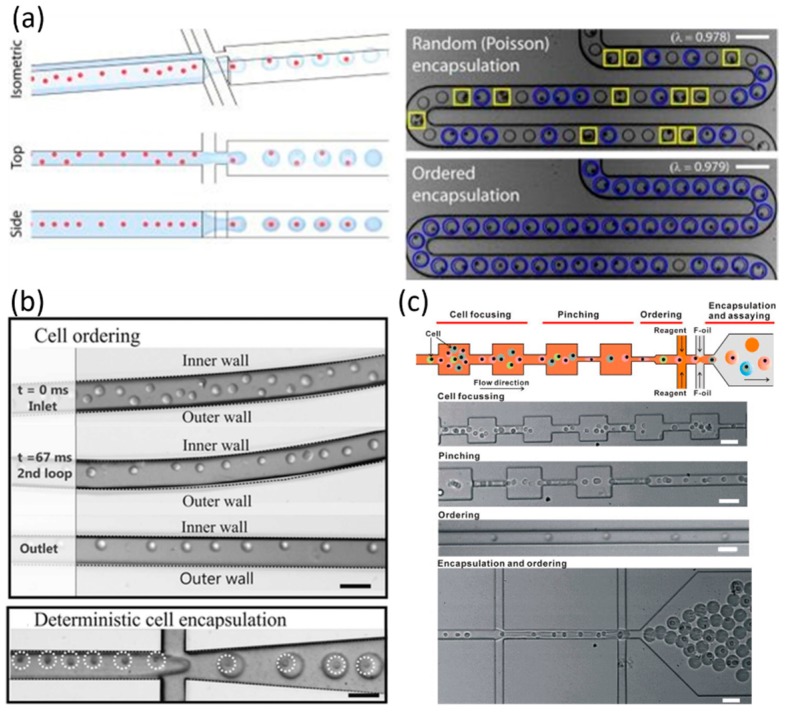
Inertial flow-based cell spacing and single-cell encapsulation using (**a**) a high aspect-ratio straight microchannel (reproduction with permission from [41]); (**b**) a curved microchannel (reproduction with permission from [42]); and (**c**) a short pinched flow channel (reproduction with permission from [43]).

Meanwhile, inertial focusing was used to evenly space single cells before the emulsification process, which can significantly improve the single-cell encapsulation efficiency in droplet microfluidics [41,42,43,44,45]. As pioneers in this field, Toner and coworkers forced a high-density suspension of cells to travel rapidly through a high aspect-ratio straight microchannel to evenly space cells, reporting a single-cell encapsulation efficiency of 80% (see Figure 3a) [41]. Furthermore, a curved microchannel was introduced to bring a second Dean force to focus cells into a single equilibrium position, with the single-cell encapsulation efficiency quantified as ~77% (see Figure 3b) [42]. Recently, a short pinched flow channel consisting of contracting and expanding chambers was also used to conduct inertial focusing along the center of the channel, enabling the encapsulation of single cells with >55% single-cell efficiencies (see Figure 3c) [43].

Although several studies have been conducted to address the issue of low efficiency of single-cell encapsulation, the optimal encapsulation efficiency was only about 80%. Thus, more studies are suggested to further address this issue from the point of technical development. However, if we view this issue from the application perspective, whether further improvements in the efficiency of single-cell encapsulation is a must is questionable. For instance, in the following sections of single-cell proteomic/genomic analysis, the relatively low trapping efficiency of single-cell confinement is indeed not a top concern.

## 4. Microfluidic Droplets Enabling Single-Cell Proteomic Analysis

Following technical developments capable of encapsulating single cells with high throughput and efficiency, the research focus was then shifted to applications in the field of single-cell proteomic and genomic analysis, which may function as integrated platforms for high-throughput screening.

**Figure 4:**
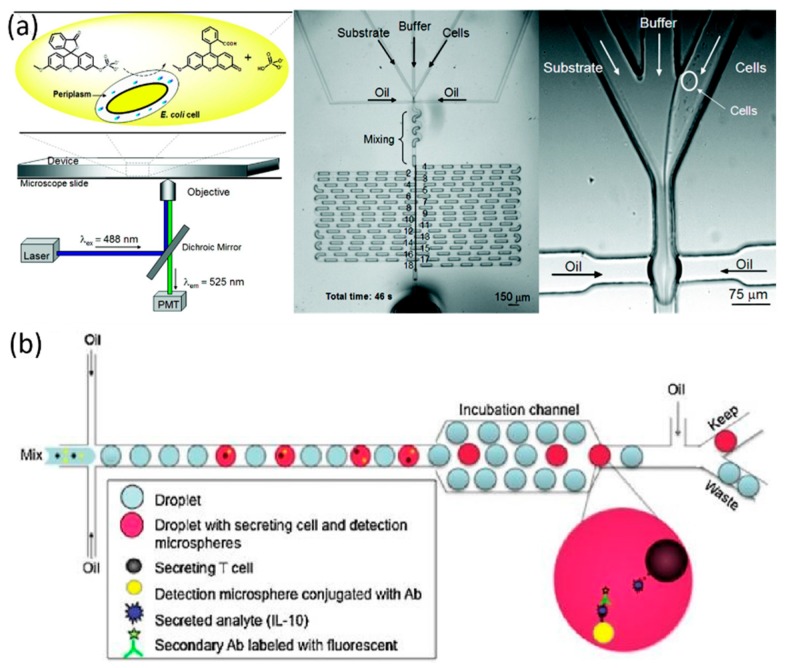
Microfluidic droplets enabling single-cell proteomic analysis. (**a**) Individual *E coli* and substrate 3-*O*-methylfluorescein-phosphates were encapsulated within single droplets where the substrates were enzymatically hydrolyzed by the target enzyme alkaline phosphatase expressed by *E coli*, leading to fluorescent detections. Reproduction with permission from [47]; (**b**) Both microspheres conjugated with capture antibodies and detection fluorescence labeled antibodies were encapsulated with single cells and the secreted IL-10 of CD4 + CD25 + regulatory T cells was captured on the microsphere surface and detected via detection antibodies, generating localized fluorescent signals on microsphere surfaces. Reproduction with permission from [58].

In the droplet microfluidics, single cells are confined within small volumes, allowing rapid accumulations of secreted metabolites to detectable levels [34,43,46,47,48,49,50,51,52,53,54,55,56,57,58,59,60,61,62,63,64,65,66,67,68,69,70] (see Table 1). The first approach was demonstrated by Hollfelder et al. to assay the activities of the enzyme alkaline phosphatase expressed by *Escherichia coli* cells. As shown in Figure 4a, individual *E coli* and substrate 3-*O*-methylfluorescein-phosphates were encapsulated within single droplets where the substrates were enzymatically hydrolyzed by the target enzyme alkaline phosphatase expressed by *E coli* cells, leading to fluorescent detections [47]. In a follow-up study, the activities of glucuronidase was quantified by encapsulating single *E. coli* with a fluorogenic reporter of 4-Methylumbelliferyl β-d-glucuronide [59]. Furthermore, the activity of receptor tyrosine kinases (RTKs) in lung cancer cells was assayed by binding surface ligands of 8-hydroxy-5-(*N*,*N*-dimethylsulfonamido)-2-methylquinoline), generating fluorescent signals [43].

As to the detection of the secreted antibodies of single cells, both microspheres conjugated with capture antibodies and detection fluorescence labeled antibodies are encapsulated with single cells. The secreted substances are captured on the microsphere surfaces, leading to the further binding of fluorescence-labeled detection antibodies, which generates localized fluorescent signals on microsphere surfaces (see Figure 4b) [58,60,64]. Using this approach, (1) secreted IL-10 of CD4 + CD25 + regulatory T cells [58]; (2) intracellular HRas-mCitrine of HEK-293 cells and actin-EGFP of MCF-7 cells [60]; and (3) secreted IL-2, IFN-γ, and TNF-α of activated T-cells [64] were assayed, respectively.

Although powerful, there are two critical concerns for single-cell proteomic analysis in droplet microfluidics. (1) The local microenvironments (e.g., nutrient levels and gas permeability) for single-cell encapsulation may be significantly different from in vivo situations and if single cells are challenged by harsh environments, the metabolites may not indicate normal activities of these cells; (2) the current approaches can only report fluorescent intensities without quantifying the absolute number of metabolites under interest due to the lack of effective calibration approaches. Since fluorescent intensities are deeply influenced by experimental setups (excitation laser intensities and geometries of the droplets), results collected by different groups cannot be effectively compared.

## 5. Microfluidic Droplets Enabling Single-Cell Genomic Analysis

**Figure 5:**
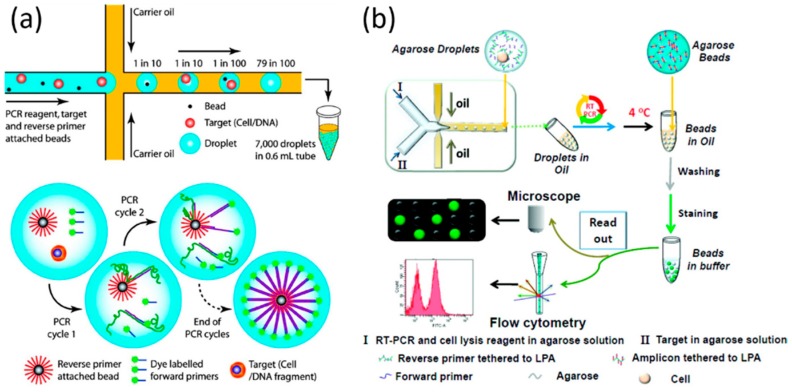
Microfluidic droplets enabling single-cell genomic analysis. (**a**) Individual cells together with primer-functionalized microbeads were encapsulated in uniform PCR mix droplets. After bulk PCR amplification, the droplets were lysed and the beads were recovered and rapidly analyzed via flow cytometry. Reproduction with permission from [71]; and (**b**) an agarose droplet-based microfluidic method for emulsification RT-PCR, where reverse primers were covalently conjugated to agarose, which functioned as the trapping matrix to replace conventional primer functionalized microbeads, resulting in high PCR efficiency (~95%). Reproduction with permission from [81].

In addition to applications in single-cell proteomic analysis, droplet microfluidics has also functioned as an effective tool enabling single-cell genomic analysis [71,72,73,74,75,76,77,78,79,80,81,82,83,84,85] (see Table 2). As pioneers in this field, Mathies et al. utilized a microfluidic droplet generator to encapsulate individual cells together with primer functionalized microbeads in uniform PCR mix droplets. After bulk PCR amplification, the droplets were lysed and the micro beads were recovered and rapidly analyzed via flow cytometry. Successful single-cell analysis of the glyceraldehyde 3 phosphate dehydrogenase gene in human lymphocyte cells and of the *gyr B* gene in bacterial *E. coli* K12 cells validated the proposed approach for performing high-throughput genetic analysis on single cells (see Figure 5a) [71].

This approach was then scaled up to an ultra-high throughput system with 96 channels in parallel to generate up to 3.4 × 10^6^ nanoliter-volume droplets per hour. Leveraging this platform, pathogenic *E. coli* O157 cells were identified in a high background of normal K12 cells, with a detection limit on the order of 1:10^5^ [75]. Furthermore, single cells and primer functionalized microbeads were confined with agarose droplets rather than aqueous droplets, which can help (1) maintain single genome fidelity during cell lysis and DNA purification; (2) improve the efficiency of emulsion PCR. Using this approach, multi-locus single-cell sequencing of the control gene β-actin and across the chromosomal translocation t (14;18), a mutation associated with 85%–90% of follicular lymphoma cases was demonstrated [79].

However, this aforementioned method requires the precision pairing of a single cell and a microbead within a single droplet, which is a challenging issue. To address this issue, Yang et al. developed a bead-free agarose droplet-based microfluidic method for emulsification PCR, where reverse primers were covalently conjugated to agarose (see Figure 5b) [74]. Since agarose functioned as the trapping matrix to replace conventional primer functionalized microbeads, the efficiency of droplet generation was increased by one to two orders of magnitude. In addition, during all of the PCR cycling temperatures, the agarose was always in the liquid form, which can effectively address the drawbacks of PCR at the solid surface of the microbeads, leading to high PCR efficiencies (~95%).

Leveraging this approach, single-cell PCR was conducted to identify a single pathogen *E. coli* O157:H7 in the high background of 100,000 excess normal K12 cells [82]. In addition, this approach was optimized to realize single-cell RT-PCR with two aqueous inlets flushed with cells and RT-PCR reagents/cell lysis buffer, respectively. Single-cell RT-PCR was successfully performed, recording a clear difference in gene expression levels of EpCAM, a cancer biomarker gene, at the single-cell level between different types of cancer cells [81].

Currently, the reported single-cell PCR in droplet microfluidics always requires the off-line amplification of interested gene sections and then droplets are flushed in the flow cytometry for further characterization. Further technical developments may integrate functional units of on-chip droplet formation, PCR or RT-PCR, as well as fluorescence detection, maximizing the multifunctional capabilities of microfluidics.

## 6. Integrated Microfluidic Droplet Systems Enabling Single-Cell Screening

The integrated microfluidic droplet systems were also developed for single-cell screening, which integrates key steps of cell encapsulation, incubation, fluorescence detection of metabolic materials, and droplet sorting relying on the fluorescent intensities [53,86,87,88,89,90,91,92,93]. As a robust working platform, this type of the integrated microfluidic system has been used to screen (1) the β-galactosidase activity of *E. coli* [86]; (2) cytotoxic effects against U937 cells [87]; (3) the horseradish peroxidase activity of yeasts [88]; (4) the effect of rifampicin against *E. coli* [89]; (5) antibody secretion capabilities of mouse hybridoma cells; and 6) xylose-overconsuming capabilities of *Saccharomyces cerevisiae* cells and L-lactate–producing capabilities of *E. coli* [92].

**Figure 6:**
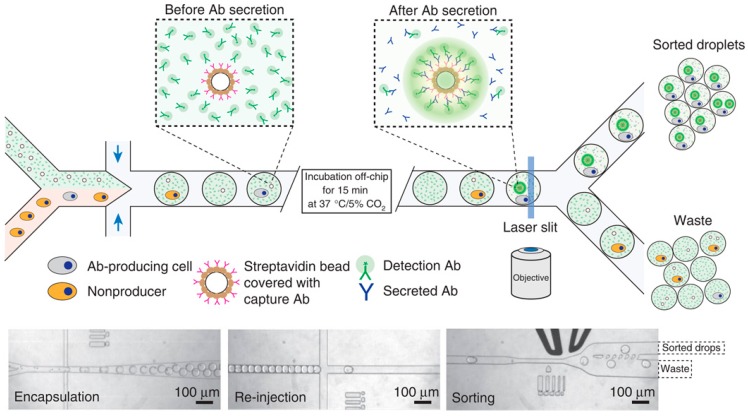
Integrated microfluidic system for single-cell screening, including key steps of cell encapsulation, incubation, fluorescence detection of metabolic molecules, and droplet sorting relying on the fluorescent intensities. Reproduction with permission from [91].

As a proof-of-concept demonstration, Griffiths et al. proposed a droplet microfluidic platform enabling the fluorescence-activated cell sorting based on enzyme activities of encapsulated single cells (see Figure 6) [86]. In this study, mixtures of *E. coli* cells, expressing either the reporter enzyme b-galactosidase or an inactive variant, were compartmentalized with a fluorogenic substrate, which were further sorted using dielectrophoresis in a fluorescence-activated manner at rates up to 300 droplets s^−1^. When the cells were encapsulated at a low density (approximately one cell for every 50 droplets), sorting was very efficient and all of the recovered cells were the active strain.

Furthermore, this approach was used to screen antibody secretion capabilities of single mouse hybridoma cells [91]. Single mouse hybridoma cells, fluorescent probes, and single beads coated with anti-mouse IgG antibodies were encapsulated in individual 50-pl droplets. Following the capture of the secreted antibodies by the micro beads, which further bind to the fluorescent probes, the fluorescence becomes localized on the beads, generating a clearly distinguishable fluorescence signal that enables droplet sorting at ~200 Hz.

## 7. Conclusions and Future Work

In this study, we summarize key developments of droplet microfluidics enabling single-cell analysis. Although significant improvements have been made within the last decade, there is still significant room left for studies in terms of throughput, multifunctionality, and absolute quantification.

Throughput is always a concern in droplet microfluidics, especially when the limited cross-sectional area of the droplet generating microchannels was taken into consideration. In the current design, multiple channels can be scaled up to generate droplets in parallel, leading to ultrahigh throughput in the formation of microdroplets. However, in the step of fluorescent detection, droplets under measurement are loaded into the microfluidic channels and detected in a serial manner, compromising the overall throughput. Future work may focus on the scaling up of the fluorescent detection units of droplet microfluidics, further improving the throughput of droplet microfluidics.

The second issue is the multi-functional capabilities of droplet microfluidics. Confinement of single cells within individual droplets is beneficial in cellular analysis without the concern of cross-contamination. However, multi-step sampling mixture and rinsing cannot be effectively conducted in droplet microfluidics, limiting functional extensions of the current microfluidic platforms. Thus, currently, only limited functional units, including cellular encapsulation, incubation, as well as fluorescent generation and detection, have been effectively integrated, and future studies may extend the functions of the current microfluidic systems by integrating more key steps including droplet fusion, division, and manipulation to the field of single-cell analysis.

In the field of single-cell droplet microfluidics, absolute quantification is a key issue, which definitely deserves more attention. Taking single-cell proteomic analysis as an example. The use of cytokine-capture beads which function as fluorescent microspots in individual droplets leads to uneven fluorescent signals in droplets, resulting in the issue of poor calibration. Without an effective calibration approach, the secreted molecules of single cells cannot be absolutely quantified and, thus, experiments from different groups cannot be compared and discussed. Thus, more studies are required to enable the absolute quantification in the field of single-cell droplets.

## Figures and Tables

**Table 1:** Key developments of microfluidic droplets enabling single-cell proteomic analysis.

Interested Proteins	Detection Mechanisms	References
β-galactosidase of mast cells	Following cellular lysis, intracellular β-galactosidase catalyzed the substrate (fluorescein di-β-d-galactopyranoside) for fluorescence detection	[34]
Yellow fluorescent protein mutant of *E. coli*	The expression of yellow fluorescent proteins was correlated with the growth status of encapsulated *E. coli*	[46]
Alkaline phosphatase of *E. coli*	Expresssed alkaline phosphatase in the cellular periplasm catalyzed the substrate (3-*O*-methylfluorescein-phosphates) for fluorescence detection	[47]
Both red fluorescent protein and alkaline phosphatase of *E. coli*	Gene expression and enzymatic activity of *E. coli* were simultaneously and continuously monitored	[50]
IL-10 of CD4+CD25+ regulatory T cells	The secreted substance captured on the microsphere surface coated with capturing antibodies and detected via the further binding of fluorescence labled detection antibodies on microsphere surfaces	[58]
Intracellular HRas-mCitrine of HEK-293 cells and actin-EGFP of MCF-7 cells	Following cell encapsulation and lysis, proteins under interest were captured on the microsphere surface coated with capturing antibodies and detected via the further binding of fluorescence labled detection antibodies on microsphere surfaces	[60]
IL-2, IFN-γ, and TNF-α of activated T-cells	Cells were encapsulated in agarose droplets together with functionalized cytokine-capture beads for subsequent binding and detection of secreted cytokines from single cells	[64]
Receptor tyrosine kinases of PC-9 cells	Binding surface ligands of 8-hydroxy-5-(*N*,*N*-dimethylsulfonamido)-2-methylquinoline) with the receptor tyrosine kinases generates fluorescent signals	[43]
Multiple proteases of MDA-MB-231, PC-9, and K-562 cells	Protease-catalyzed multi-color Förster resonance energy transfer based enzymatic substrates, enabling the simultaneous measurement of six proteases	[70]

**Table 2:** Key developments of microfluidic droplets enabling single-cell genomic analysis.

Interested Gene Sections	Working Mechanisms	References
GAPDH gene of lymphocyte cells and gyr B gene of *E. coli*	A single cell and a primer functionalized microbead were encapsulated in droplets, followed by bulk PCR, droplet lysis, and bead analysis in flow cytometry	[71]
KI#128 island on the *E. coli* K12 and OI#43 island on the *E. coli* O157 cells	96 channels were used to generate up to 3.4 × 10^6^ nanoliter-volume droplets per hour, identifying rare pathogenic *E. coli* O157 cells (1:10^5^ cells)	[75]
Chromosomal translocation t(14;18) of follicular lymphoma cells	Agarose droplets were formed to encapsulate cells and primer-functionalized microbeads, maintaining genome fidelity during cell lysis and DNA purification, leading to efficient PCR and subsequent gene sequencing	[79]
KI#128 island on the *E. coli* K12 and OI#43 island on the *E. coli* O157 cells	An agarose droplet was formed to encapsulate single cells and PCR mix with reverse primers covalently conjugated to agarose	[82]
Gene expression of EpCAM from	An agarose droplet was formed to encapsulate single cells and RT-PCR mix with primers covalently conjugated to agarose	[82]
